# Supplementary material for: Signals of Potential Species Associations Offer Clues about Community Organisation of Stream Fish across Seasons
Source: Animals (Basel). 2022 Jul 3;12(13):1721. doi: 10.3390/ani12131721 (PMC9265093; doi:10.3390/ani12131721)
Supplement: Supplementary file 1 [file animals-12-01721-s001.zip › animals-1718420-supplementary.pdf]

Table S1. Abbreviation and feeding habits of the fish species in the Qiupu River

Species	Abbreviation	Feeding habits
Acheilognathus barbatulus	A. barbatulus	planktivore
Acheilognathus chankaensis	A. chankaensis	planktivore
Acrossocheilus fasciatus	A. fasciatus	planktivore
Aristichthys nobilis	A. nobilis	planktivore
Abbottina rivularis	A. rivularis	benthivore
Acheilognathus taenianalis	A. taenianalis	planktivore
Culter alburnus	C. alburnus	piscivore
Carassius auratus	C. auratus	omnivore
Cyprinus carpio	C. carpio	omnivore
Cultrichthys erythropterus	C. erythropterus	piscivore
Culter mongolicus	C. mongolicus	piscivore
Coilia nasus	C. nasus	omnivore
Cobitis rarus	C. rarus	benthivore
Culter dabryi	C.dabryi	piscivore
Hemibarbus labeo	H. labeo	benthivore
Hemiculter leucisculus	H. leucisculus	omnivore
Hypophthalmichthys molitrix	H. molitrix	planktivore
Leptobotia guilinensis	L. guilinensis	omnivore
Liobagrus styani	L. styani	omnivore
Pelteobagrus vachelli	P. vachelli	benthivore
Misgurnus anguillicaudatus	M. anguillicaudatus	omnivore
Macropodus chinensis	M. chinensis	planktivore
Microphysogobio fukiensis	M. fukiensis	benthivore
Mylopharyngodon piceus	M. piceus	benthivore
Megalobrama skolkovii	M. skolkovii	omnivore
Micropercops swinhonis	M. swinhonis	omnivore
Opsariichthys bidens	O. bidens	omnivore
Odontobutis obscurus	O. obscurus	piscivore
Paramisgurnus dabryanus	P. dabryanus	omnivore
Pseudohemiculter dispar	P. dispar	omnivore
Pseudorasbora elongata	P. elongata	planktivore
Pelteobagrus fulvidraco	P. fulvidraco	benthivore
Pelteobagrus nitidus	P. nitidus	benthivore
Phoxinus oxycephalus	P. oxycephalus	benthivore
Pseudorasbora parva	P. parva	benthivore
Pseudobrama simoni	P. simoni	planktivore
Pseudobagrus truncatus	P. truncatus	benthivore
Rhinogobius giurinus	R. giurinus	omnivore
Squalidus argentatus	S. argentatus	benthivore
Silurus asotus	S. asotus	piscivore
Siniperca chuatsi	S. chuatsi	piscivore

**Species**	**Abbreviation**	**Feeding habits**
Saurogobio dabryi	S. dabryi	benthivore
Sarcocheilichthys nigripinnis	S. nigripinnis	benthivore
Squalidus nitens	S. nitens	benthivore
Sarcocheilichthys parvus	S. parvus	benthivore
Siniperca scherzeri	S. scherzeri	piscivore
Sarcocheilichthys sinensis	Sa. sinensis	omnivore
Sinobdella sinensis	Si. sinensis	omnivore
Hemiculter bleekeri	H. bleekeri	omnivore
Vanmanenia stenosoma	V. stenosoma	benthivore
Xenocypris argentea	X. argentea	planktivore
Xenocypris microlepis	X. microlepis	planktivore
Zacco platypus	Z. platypus	omnivore

Table S2. Fish richness and abundance for the three stream orders in the dry and wet seasons of the Qiupu River

	**Second_order**	**Third_order**	**Fourth_order**
**Dry season**			
Fish richness	14	21	26
Fish abundance	548	171	130
**Wet season**			
Fish richness	18	25	31
Fish abundance	301	51	327

Table S3. Average values and ranges of the environmental covariates for the three stream orders in the wet season of the Qiupu River

Environmental factors	**Second_order**			**Third_order**			**Fourth_order**		
	Max	Min	Ave	Max	Min	Ave	Max	Min	Ave
Elevation (m)	132.00	59.00	87.75	52.00	5.00	31.00	5.00	0.00	1.63
Dissolved oxygen (mg/L)	8.38	5.40	6.85	4.79	19.47	11.16	17.74	9.19	12.44
Water temperature (°C)	24.50	21.20	22.98	24.00	22.20	23.28	26.60	23.10	24.90
Conductivity (μs/cm)	253.40	203.40	232.99	172.10	271.40	209.80	213.60	199.80	207.61
Stream width (m)	81.40	21.30	62.19	157.00	93.00	113.93	214.00	130.00	166.63
Water depth (m)	71.00	20.00	47.63	150.00	50.00	85.00	210.00	80.00	150.00
Current velocity (m/s)	1.50	0.10	0.70	0.50	0.00	0.25	0.50	0.20	0.38
Boulder (%)	40.00	0.00	7.50	0.00	0.00	0.00	0.00	0.00	0.00
Cobble (%)	30.00	0.00	8.75	20.00	0.00	12.50	0.00	0.00	0.00
Pebble (%)	40.00	0.00	25.00	40.00	10.00	25.00	30.00	0.00	6.25
Gravel (%)	70.00	0.00	37.50	50.00	20.00	30.00	60.00	40.00	48.75
Sand (%)	60.00	0.00	21.25	40.00	20.00	32.50	60.00	20.00	45.00
Forest (%)	0.99	0.39	0.66	0.87	0.01	0.48	0.24	0.00	0.10
Grassland (%)	0.41	0.00	0.12	0.56	0.00	0.17	0.42	0.00	0.08
Urban (%)	0.01	0.00	0.00	0.00	0.00	0.00	0.01	0.00	0.00
Agriculture (%)	0.44	0.00	0.22	0.46	0.12	0.36	1.00	0.33	0.80
Waterbodies (%)	0.00	0.00	0.00	0.00	0.00	0.00	0.07	0.00	0.02

Table S4. Average values and ranges of the environmental covariates for the three stream orders in the dry season of the Qiupu River

Environmental factors	**Second_order**			**Third_order**			**Fourth_order**		
	Max	Min	Ave	Max	Min	Ave	Max	Min	Ave
Elevation (m)	122.00	54.00	87.25	28.00	5.00	14.50	5.00	0.00	1.63
Dissolved oxygen (mg/L)	11.75	6.92	8.64	11.21	9.34	10.29	8.00	5.87	6.74
Water temperature (℃)	12.10	7.40	10.68	13.40	10.10	12.03	13.70	10.20	11.04
Conductivity (μs/cm)	254.80	129.90	198.58	297.60	176.40	259.60	335.30	275.10	299.00
Stream width (m)	41.00	20.00	29.88	59.00	30.00	48.00	66.00	41.00	52.88
Water depth (m)	66.00	26.00	38.25	100.00	60.00	59.75	120.00	55.00	86.25
Current velocity (m/s)	0.30	0.10	0.14	0.40	0.00	0.20	0.50	0.30	0.41
Boulder (%)	0.40	0.00	0.08	0.00	0.00	0.00	0.00	0.00	0.00
Cobble (%)	0.30	0.00	0.09	0.20	0.00	0.13	0.00	0.00	0.00
Pebble (%)	0.40	0.00	0.25	0.40	0.10	0.25	0.30	0.10	0.06
Gravel (%)	0.70	0.00	0.38	0.50	0.20	0.30	0.60	0.40	0.49
Sand (%)	0.60	0.00	0.21	0.40	0.20	0.33	0.60	0.20	0.45
Forest (%)	0.99	0.39	0.66	0.88	0.01	0.48	0.24	0.00	0.10
Grassland (%)	0.41	0.00	0.12	0.56	0.00	0.17	0.42	0.00	0.08
Urban (%)	0.01	0.00	0.00	0.00	0.00	0.00	0.01	0.00	0.00
Agriculture (%)	0.44	0.00	0.22	0.47	0.12	0.36	1.00	0.33	0.80
Waterbodies (%)	0.00	0.00	0.00	0.00	0.00	0.00	0.07	0.00	0.02

Table S5. Model fit diagnostics and prediction errors for each Poisson log–normal (PLN) models in dry season.

Model	BIC	RMSE	BIC	RMSE	BIC	RMSE
**Dry season**	**Second–order**		**Third–order**		**Fourth-order**	
Site	-268.8442	0.5791385	-263.1233	0.2006302	-557.0691	0.3389599
Site + Elevation	-267.7142	0.5671455	-256.4282	0.2353072	-566.0555	0.3630749
Site + Dissolved oxygen	-272.9281	0.5993287	-264.8085	0.1634944	-572.1259	0.2911683
Site + Water temperature	-275.0192	0.7513443	-271.4026	0.4297821	-579.9218	0.3001027
Site + Conductivity	-361.3449	1.3954962	-306.2135	0.8645403	-633.7147	0.5754614
Site + Stream width	-276.2094	0.5734773	-304.7802	0.9764922	-632.0535	0.5670329
Site + Water depth	-273.2998	0.7123752	-300.6710	0.9423611	-636.8832	0.6394525
Site + Current velocity	-276.4271	0.6066771	/	/	/	/
Site + Boulder	-271.9447	0.6105734	-266.5269	0.2675446	/	/
Site + Cobble	-270.5493	0.6821490	-261.6769	0.1999360	-583.8409	0.3193222
Site + Pebble	-273.2037	0.6334642	-265.4749	0.1974774	-580.7233	0.3027647
Site + Gravel	-294.2110	0.6603473	-265.1395	0.1949509	-578.0253	0.3556943
Site + Sand	-269.4481	0.7600104	-268.4440	0.2737420	-585.0260	0.3107738

Table S6. Model fit diagnostics and prediction errors for each Poisson log–normal (PLN) models in wet season.

Model	BIC	RMSE	BIC	RMSE	BIC	RMSE
**Wet season**	**Second–order**		**Third–order**		**Fourth-order**	
Site	-379.2315	0.6155388	-323.5700	0.11084218	-825.6100	0.3049280
Site + Elevation	-424.2716	0.7086136	-336.1901	0.19277331	-828.2203	0.4026995
Site + Dissolved oxygen	-385.0173	0.4874386	-337.2866	0.18944651	-820.5501	0.2530538
Site + Water temperature	-393.5658	0.6282974	-338.9942	0.25709896	-850.2098	0.3802063
Site + Conductivity	-440.8542	0.6392205	-351.3117	0.54155741	-905.6762	0.7244338
Site + Stream width	-430.1546	0.6645610	-328.8042	0.12907598	-907.9380	0.7077361
Site + Water depth	-390.7146	0.6672273	-331.6417	0.09927637	-903.4869	0.6838925
Site + Current velocity	-388.4087	0.5969755	-330.6973	0.03219890	/	/
Site + Boulder	-387.7962	0.6576995	-331.6027	0.05263872	/	/
Site + Cobble	-389.2447	0.5959866	-332.4193	0.08411214	-841.6615	0.2958145
Site + Pebble	-386.3100	0.5957144	/	/	-840.5596	0.3267319
Site + Gravel	-390.4531	0.5676425	-331.7339	0.08035994	-846.2166	0.3488692
Site + Sand	-386.1569	0.5398407	-331.8530	0.12287592	-845.3906	0.3303147

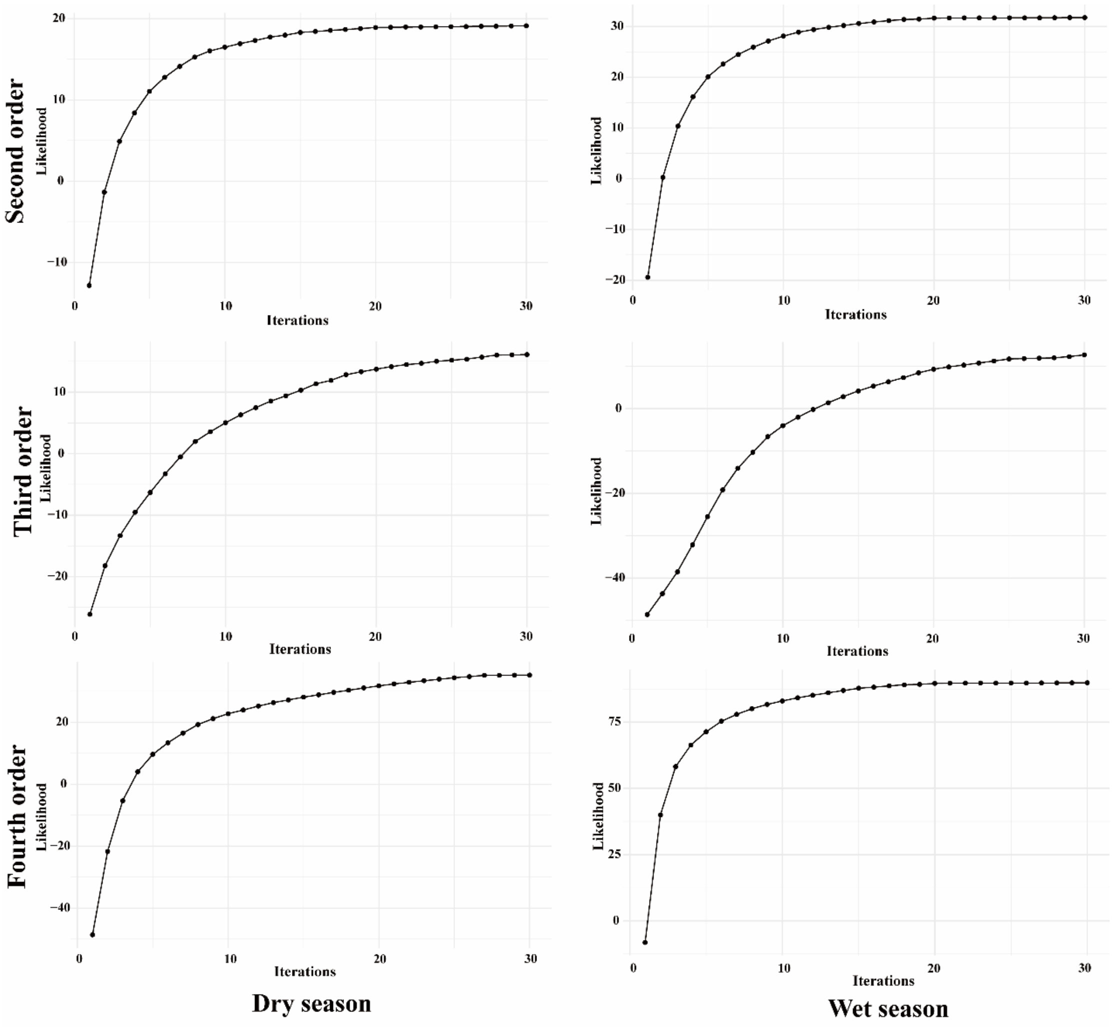

Figure S1. Plots of likelihood fitted curve of each network
